# LINC00312/YBX1 Axis Regulates Myofibroblast Activities in Oral Submucous Fibrosis

**DOI:** 10.3390/ijms21082979

**Published:** 2020-04-23

**Authors:** Chuan-Hang Yu, Chih-Yuan Fang, Cheng-Chia Yu, Pei-Ling Hsieh, Yi-Wen Liao, Lo-Lin Tsai, Pei-Ming Chu

**Affiliations:** 1 School of Dentistry, Chung Shan Medical University, Taichung 40201, Taiwan; tao2008@csmu.edu.tw (C.-H.Y.); ccyu@csmu.edu.tw (C.-C.Y.); rabbity0225@gmail.com (Y.-W.L.); 2 Department of Dentistry, Chung Shan Medical University Hospital, Taichung 40201, Taiwan; 3 Institute of Oral Sciences, Chung Shan Medical University, Taichung 40201, Taiwan; 4 School of Dentistry, College of Oral Medicine, Taipei Medical University, Taipei 110, Taiwan; 100044@w.tmu.edu.tw; 5 Division of Oral and Maxillofacial Surgery, Department of Dentistry, Wan Fang Hospital, Taipei Medical University, Taipei 116, Taiwan; 6 Department of Anatomy, School of Medicine, China Medical University, Taichung 404, Taiwan; plhsieh@mail.cmu.edu.tw

**Keywords:** LINC00312, YBX1, oral submucous fibrosis, myofibroblast

## Abstract

Oral submucous fibrosis (OSF) has been recognized as a precancerous disorder in the oral cavity. Great effort has been made to inhibit the malignant progression of OSF over the past decades, but the cure of this fibrosis disease has not been discovered. In the present study, we found that a long noncoding RNA, LINC00312, was upregulated in OSF tissues, and positively associated with several fibrosis factors, such as α-SMA, type I collagen, and fibronectin. As such, we sought to investigate the role of LINC00312 in OSF progression and identify its interacting factor that mediated oral fibrogenesis. Our results showed that the inhibition of LINC00312 downregulated the myofibroblast activities, including collagen gel contractility, transwell migration, and wound healing, as well as the gene expression of myofibroblast markers. We verified that YBX1 was a downstream factor of LINC00312 and revealed that the downregulation of YBX1 repressed the gene expression of α-SMA and p-Smad2 along with the reduced myofibroblast phenotypes. Most importantly, we demonstrated that the LINC00312-induced myofibroblast activities were reverted by the knockdown of YBX1, suggesting that the LINC00312-mediated myofibroblast transdifferentiation was through YBX1. Collectively, our findings revealed that the LINC00312/ YBX1 axis may serve as a target for the development of therapies against OSF.

## 1. Introduction

Oral submucous fibrosis (OSF) is a chronic fibrotic disease and has been listed as one of the potentially malignant disorders of the oral cavity (OPMD). One of the recent epidemiology studies has shown that the overall prevalence of OPMD was around 4.47%, and OSF was the one with the highest prevalence (4.96%) [1]. Among the subtypes of OPMD, OSF has the third-highest malignant transformation rate [2]. Nevertheless, it is still challenging to detect lesions undergoing malignant transformation using the current noninvasive diagnostic devices due to some difficulties, such as interobserver variability [3]. Since the efficacy of the most recent diagnostic methods to monitor oral lesions was not ideal and the prognosis of patients with recurrent or metastatic oral cancer treated with chemotherapy remains poor [4], it is imperative to prevent or delay the progression of OSF into oral cancer and decipher the etiological mechanisms of OSF. Although cigarette smoking had a substantial role in the occurrence of OSF, the habit of betel quid chewing has been recognized to significantly contribute to the risks of OSF development [5] and be one of the reasons that OSF was more commonly reported in South Asia [1]. The major alkaloid in betel quid, arecoline, has been proven to induce myofibroblast transdifferentiation from human buccal mucosal fibroblasts (BMFs) with the increased expression of α-smooth muscle actin (α-SMA) [6], and the activated myofibroblasts have been considered as the key pathogenic cells that conferred to excessive deposition of extracellular matrix [7]. Further elucidation of the molecular mechanism underlying the activation of myofibroblasts may improve the development of effective therapy for OSF patients.

After they were discovered, emerging evidence has suggested that noncoding RNAs (ncRNAs) were essential regulators of cellular signaling even if they do not possess the protein-coding ability. In particular, long noncoding RNAs (lncRNAs) have been known to be a type of ncRNAs that are longer than 200 nucleotides and a couple of studies have revealed that lncRNAs may participate in the pathogenesis of OSF [8,9]. One of the recent studies regarding the expression profile associated with malignant progression of OSF has revealed that there were around 700 aberrantly expressed lncRNAs that may be involved in the regulation of different stages of OSF development [10]. As a result, it is crucial to select the abnormally expressed lncRNAs and investigate their role in the activation of myofibroblasts and examine whether targeting these lncRNAs could result in a promising effect.

In the present study, we unraveled that LINC00312 was upregulated in OSF tissues. Aside from revealing its association with various fibrosis factors, we also examined its effect on the activation of the fibrotic BMFs (fBMFs), including the phenotypes and the expression of fibrosis markers. Moreover, we used the Rtool website to predict the potential interacting factor of LINC00312 and identified that YBX1 was a putative interactome of LINC00312. After assessing the functional role of YBX1 in myofibroblast activities, our results verified that LINC00312-mediated transdifferentiation of BMFs was via the upregulation of YBX1.

## 2. Results

### 2.1. LINC00312 is Upregulated in OSF Tissues and Associated with Fibrosis Markers

**Figure 1:**
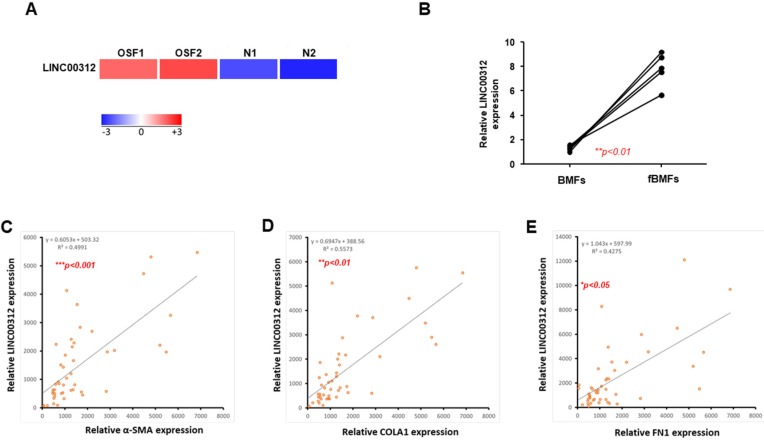
The expression of LINC00312 is upregulated in oral submucous fibrosis (OSF) tissues and positively correlated with fibrosis markers. (**A**) RNA-seq analysis revealed that LINC00312 was overexpressed in OSF tissues compared to the normal buccal mucosa. (**B**) The relative expression level of LINC00312 were higher in human fibrotic buccal mucosal fibroblasts (fBMFs, *n* = 5) relative to human normal buccal mucosal fibroblasts (BMFs, *n* = 5). The expression of LINC00312 was positively correlated with (**C**) ACTA2 (α-SMA), (**D**) COL1A1 (alpha 1 type I collagen), and (**E**) FN1 (fibronectin) expressions in OSF samples from using qRT-PCR and Pearson’s correlation coefficient.

To detect the key lncRNA involved in the fibrogenesis, the two collected normal and OSF tissues were subjected to RNA sequencing analysis. Our results revealed that LINC00312 was one of the lncRNAs with differential expression and the expression of LINC00312 was elevated in OSF tissues compared to the normal counterparts (Figure 1A). The expression of LINC00312 was positively associated with myofibroblast markers using Pearson’s correlation analysis (Supplementary Figure S1A). Our results also showed that LINC00312 was overexpressed in fBMFs compared to normal BMFs (Figure 1B). We observed that the expression of LINC00312 was positively related to several myofibroblast markers, including α-SMA (Figure 1C), alpha 1 type I collagen (Figure 1D), and fibronectin (Figure 1E). These findings suggested that LINC00312 may contribute to oral fibrogenesis.

### 2.2. Suppression of LINC00312 in fBMFs Downregulates the Myofibroblast Features

**Figure 2:**
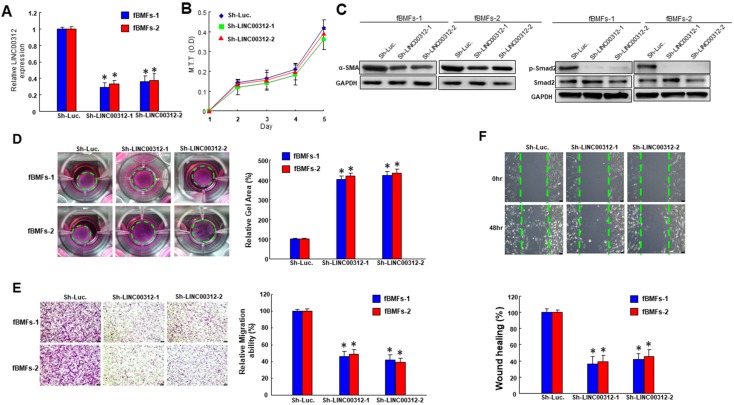
The knockdown of LINC00312 ablates myofibroblast features in fBMFs. (**A**) The silencing effect of LINC00312 in fBMFs was validated by qRT-PCR analysis. (**B**) The proliferation rate of sh-Luc or LINC00312-knockdown fBMFs was analyzed by an MTT assay. (**C**) The silencing of LINC00312 repressed the expression level of myofibroblast markers (α-SMA and p-Smad2), (**D**) collagen gel contraction, (**E**) transwell migration, and (**F**) wound healing abilities in two lines of patient-derived fBMFs with a lentiviral-mediated LINC00312 knockdown. Experiments were repeated three times and representative results were shown. Results were presented as means ± SD. * *p* < 0.05 compared with the sh-Luc control.

In order to investigate the effect of LINC00312 on the myofibroblast transdifferentiation, we employed a small hairpin to silence the expression of LINC00312 in two lines of OSF patient-derived fibrtic buccal mucosal fibroblasts (fBMFs) (Figure 2A). The proliferation rate was not changed in LINC00312-knockdown fBMFs (Figure 2B). The expression of myofibroblast and fibrosis markers, α-SMA and p-Smad2 (Figure 2C and Supplementary Figure S1B) was downregulated. We also found that the ability of collagen gel contraction was reduced (Figure 2D). In addition, the cell motility of fBMFs toward a chemo-attractant gradient was diminished in the LINC00312-reduced fBMFs (Figure 2E). Moreover, the wound healing capacity of fBMFs was downregulated after a silence of LINC00312 (Figure 2F). These results indicated that the expression of LINC00312 affected the phenotypes and markers of myofibroblasts.

### 2.3. YBX1 is a Putative Target of LINC00312 and Increased in the OSF Specimens

**Figure 3:**
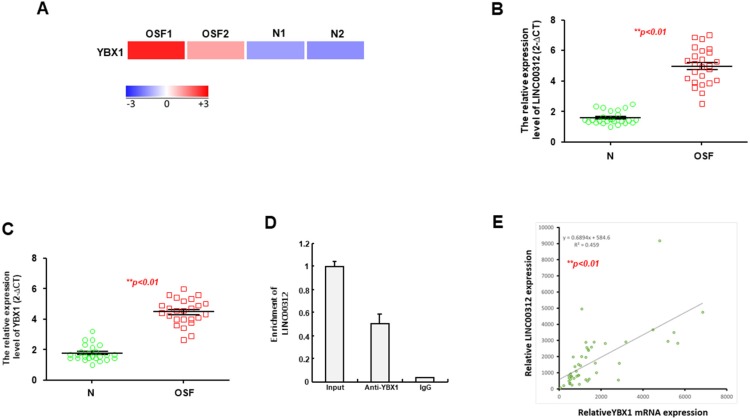
The Y-box binding protein 1 (YBX1) is a putative interacting factor of LINC00312. (**A**) YBX1 has been identified as one of the predicted interactomes of LINC00312 using the Rtool website. As shown in the lower panel, YBX1 was upregulated in the OSF tissues compared to the normal buccal mucosa by RNA-seq analysis. The increased expression of LINC00312 (**B**) and YBX1 (**C**) relative to the normal tissue in OSF specimens (*n* = 25) were validated using qRT-PCR analysis. (**D**) The enrichment of LINC00312 was enriched in the anti-YBX1 group, compared to the control IgG group by an RIP assay. (**E**) The expression of LINC00312 was positively correlated with YBX1 expressions in OSF samples using Pearson’s correlation coefficient analysis.

In an attempt to predict the interacting factors that were regulated by LINC00312, we used the Rtools web server and selected Y-box binding protein 1 (YBX1) for further examination as RNA sequencing analysis showed that YBX1 was upregulated in OSF tissues compared to the normal buccal mucosa using the lower panel (Figure 3A). The increased expression levels of LINC00312 (Figure 3B) and YBX1 (Figure 3C) in OSF specimens were validated using qRT-PCR analysis. We carried out an RIP assay using a YBX1-specific antibody followed by qRT-PCR with primers specific for LINC00312 to verify their interaction. As expected, LINC00312 was enriched in the anti-YBX1 group, compared to the control IgG group (Figure 3D). Moreover, we observed a positive correlation between the expression of LINC00312 and YBX1 (Figure 3E), which also was in favor of our hypothesis that YBX1 interacts with LINC00312.

### 2.4. Inhibition of YBX1 ameliorates the characteristics of fBMFs

**Figure 4:**
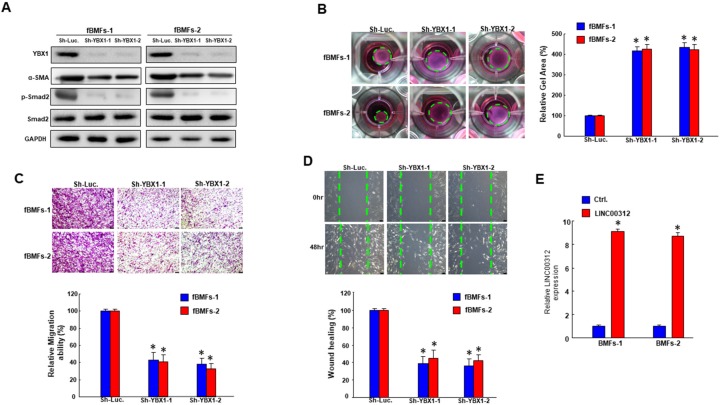
Downregulation of YBX1 in fBMFs diminishes the expression of fibrosis-associated markers and the myofibroblast activities. (**A**) Knockdown efficiency of YBX1 and the inhibited expression of fibrosis-associated markers (α-SMA and p-Smad2) were confirmed by Western blot following the silencing of YBX1. Downregulation of YBX1 in fBMFs repressed the myofibroblast activities, including collagen gen contractility (**B**), migration (**C**), and wound healing (**D**) capacities. Results are means ± SD of triplicate samples from three experiments. (**E**) The overexpression effect of LINC00312 in BMFs was validated by qRT-PCR analysis. * *p* < 0.05 compared to the Sh-Luc group.

To investigate whether LINC00312 modulated the features of myofibroblasts through YBX1, we sought to examine the effect of YBX1 on myofibroblast activities. Our results showed that the knockdown of YBX1 inhibited the expression of α-SMA and phosphorylated Smad2 (Figure 4A and Supplementary Figure S1C). In addition to the reduced expression of fibrosis markers, collagen gel contractility of fBMFs was decreased as well (Figure 4B). In addition, the transwell migration ability (Figure 4C) and wound healing abilities (Figure 4D) were both declined. Altogether, these findings suggested that the expression of YBX1 regulated the myofibroblast activities of fBMFs.

### 2.5. LINC00312 Enhances the Myofibroblast Activities via YBX1

**Figure 5:**
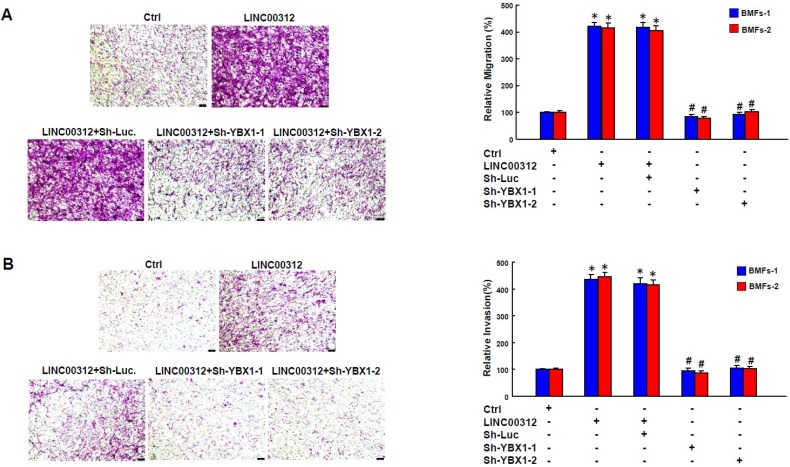
The knockdown of YBX1 reverses the LINC00312-increased migration and invasion capabilities in BMFs. Transwell migration (**A**) and invasion (**B**) of BMFs transfected with the indicated plasmids. Data were shown as the mean ± SD. *p < 0.05 compared to the control group. # *p* < 0.05 compared to the LINC00312+Sh-Luc group.

**Figure 6:**
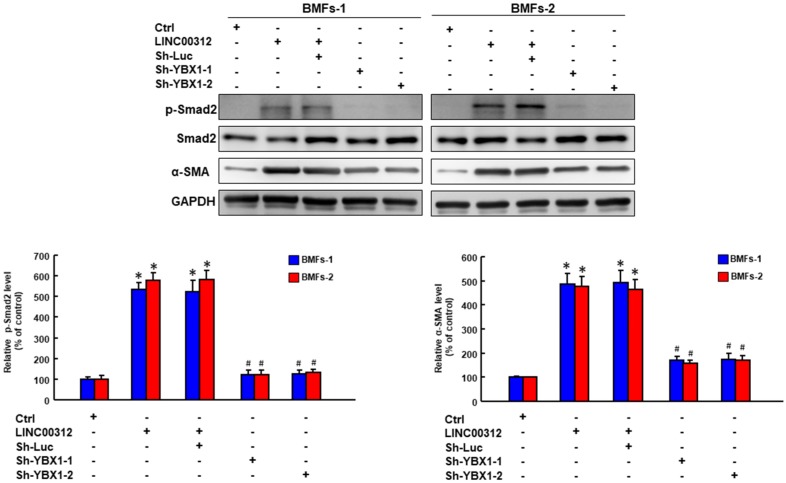
The knockdown of YBX1 reverses the LINC00312-increased α-SMA and p-SMAD2 in BMFs. The expression levels of α-SMA and p-SMAD2 as indicated transfections presented by the Western blotting analysis. *p < 0.05 compared to the control group. # *p* < 0.05 compared to the LINC00312+Sh-Luc group.

After confirming the effect of YBX1 and LINC00312 on myofibroblast activities, the last step was to verify that LINC00312 regulated the activation of myofibroblasts through YBX1. We demonstrated that overexpression of LINC00312 (Figure 4E) increased the transwell migration ability of BMFs, while the knockdown of YBX1 reverted this effect (Figure 5A). Likewise, the elevation of LINC00312 in BMFs induced the invasion capacity, whereas the silencing of YBX1 reversed the LINC00312-enhanced invasion ability (Figure 5B). We found that YBX1 suppression reverted the induced p-Smad2 and α-SMA by LINC00312 overexpression in BMFs (Figure 6). Collectively, these results showed that the increased expression of LINC00312 stimulated the transdifferentiation of BMFs into myofibroblasts via interacting with YBX1.

## 3. Discussion

LINC00312, also known as NAG7, is a long intergenic ncRNA located on chromosome 3p25.3. It was first reported in the nasopharyngeal carcinoma (NPC) and this downregulated gene has been found to be a transmembrane protein containing a protein kinase C phosphorylation site and a myristyl site [11]. It has been shown that LINC00312 has a double effect in NPC as LINC00312 not only possessed the inhibitory effect on proliferation but also promoted the invasion of NPC cells. LINC00312 has been demonstrated to be a negative regulator of the estrogen receptor α, and activates the JNK2/AP-1/MMP1 pathway to enhance the invasion of NPC [12]. In other types of cancers, overexpression of LINC00312 was able to inhibit the migration and invasion of bladder and thyroid cancer cells by targeting miR-197-3p [13,14]. LINC00312 also has been shown to diminish the aggressiveness of thyroid cancer through suppression of the PI3K/Akt pathway [15]. In addition, LINC00312 was found to inhibit colorectal cancer cells by binding to miR-21, therefore, increasing PTEN [16]. Another study showed that LINC00312 could downregulate cyclin B1 and induce G2-M cell cycle arrest in hepatocellular carcinoma cells, which downregulated cell proliferation and tumor progression in vivo [17]. These studies showed that LINC00312 may affect multiple pathways, molecules, and miRs (one type of small ncRNA) to regulate cancer progression. As for its upstream modulator, only HOXA5 has been reported to bind to the promoter of LINC00312 and upregulate the expression of LINC00312, leading to increased apoptosis in nonsmall cell lung cancer (NSCLC) [18]. Collectively, these results suggested that LINC00312 was inclined to play a tumor suppressor role in the majority of cancers. However, its effect on fibrogenesis has not been investigated yet.

Unlike its suppressive role in various cancers, we observed an upregulation of LINC00312 in OSF tissues. In addition, the expression of LINC00312 was positively related to several fibrosis factors, including α-SMA, type I collagen α1, and fibronectin. α-SMA has been shown to upregulate the fibroblast contractile activity [19] and represent a hallmark of the myofibroblast. Type I collagen has been known as the primary component of the excess deposition of collagens in fibrosis. Moreover, it has been shown that the production of type I collagen, especially α 1(I) chains, was increased in myofibroblasts derived from OSF tissues [20]. The accumulation of fibronectin was another contributing factor that led to OSF [21]. These results suggested that LINC00312 may contribute to the fibrosis and modulate the progression of OSF. In association with our hypothesis, knockdown of LINC00312 reduced the myofibroblast activities, including collagen gel contractility and migration. In light of the function of myofibroblasts to migrate to the injured site and close the wound, we chose these two phenotypes (migration and wound healing capacities) to examine the effect of LINC00312. The result from Western blot also demonstrated a downregulated expression of α-SMA in fBMFs with sh-LINC00312, indicating that modulation of LINC00312 was able to inhibit the activation of myofibroblasts. Subsequently, we aimed to elucidate the interacting factor of LINC00312 by screening from the Rtool website and selected YBX1 as our target. We showed that the expression levels of LINC00312 and YBX1 were both increased in OSF specimens and there was a positive correlation between these two molecules. Moreover, LINC00312 directly binds to YBX1 by an RIP assay. We also demonstrated that the increased myofibroblast activities induced by LINC00312 overexpression were counteracted by sh-YBX1, which suggested that the LINC00312-mediated myofibroblast activation required YBX1. These findings were consistent with a previous report showing that LINC00312 could directly bind to YBX1 and induce lung adenocarcinoma migration and vasculogenic mimicry [22].

YB1, encoded by the YBX1 gene, is a member of the cold shock protein and has been known as a negative regulator of collagen expression by directly binding to an interferon-gamma response element within the COL1A2 promoter [23] or through a cross-talk with the TGFβ/Smad signaling pathway [24]. Overexpression of YB1 suppressed the endogenous COL1A1 expression and collagen protein production in normal rat kidney epithelial cells, as well [25]. Although these results suggested that YB1 possessed the anti-fibrosis effect, the upregulation of YB1 was found in the liver fibrosis tissues and demonstrated the deposition promotion of excess extracellular matrix [26]. In our results, we also observed the overexpression of YBX1 in OSF tissues. In fact, accumulating evidence has revealed that YB1 played both pro- and anti-fibrotic roles based on its subcellular localization. YB1 was mostly localized in the cytoplasm, and nuclear translocation of YB1 has been proven to ameliorate experimental hepatic fibrosis in mice [27]. Furthermore, YB1 was involved in the TGFβ-mediated upregulation of α-SMA and it dissociated from the α-SMA enhancer DNA in the presence of TGFβ or its Smad 2, 3, 4 coregulatory mediators in human fibroblasts [28]. Aside from being able to form a complex with Smad3 [24] and interact with α-SMA [28], YB1 also has been shown to have a positive feedback loop with Smad2 [26]. Xiong et al. showed that Smad2 functioned as a transcription factor that triggered the YB1 promoter, while phosphorylated YB1 stabilized Smad2 by attenuating its ubiquitination [26]. In our results, we demonstrated that the silencing of YBX1 resulted in the downregulation of α-SMA and phosphorylated Smad2, which were in conformity with the previous findings showing that YBX1 coordinated the activation of α-SMA and Smad2. Moreover, we demonstrated that the repression of YBX1 in fBMFs reduced the myofibroblast activities, suggesting that targeting YBX1 may be an option to relieve the symptoms of OSF.

One limitation of the current study was a lack of in vivo research. Currently, there was no well-established animal model to mimic the pathogenesis of OSF. These methods were either time consuming [29] or not suitable for studying fibrosis in the oral mucosae as the induced fibrosis occurred in the back [30]. Further studies are needed to examine the effect of targeting YBX1 on relief OSF symptoms in vivo.

## 4. Materials and Methods

### 4.1. Cell Culture for Buccal Mucosa Fibroblasts (BMFs) and Fibrotic Buccal Mucosa Fibroblasts (fBMFs)

All procedures were conducted under the approval from the Institutional Review Board of Chung Shan Medical University Hospital, Taichung, Taiwan (approval number: CSMUH No. CS2-17018; approval date: 15 June 2017). Primary normal buccal mucosa fibroblasts (BMFs) and fibrotic buccal mucosa fibroblasts (fBMFs) were isolated from normal human buccal oral mucosa and OSF lesions, respectively, with written informed consent from patients. These patient-derived fBMFs exhibited myofibroblasts characteristics and have been used in several studies [6,31]. Cells were cultured as previously described [6], and passaged routinely at 90% confluence. BMFs or fBMFs were migrated from the tissue margin and began to proliferate in a dish containing 10% fetal bovine serum in a DMEM medium. Cell cultures between the third and sixth passages were used in this study.

### 4.2. Tissues Acquirements

All procedures of tissues acquirements have followed the tenets of the Declaration of Helsinki and were approved by the Institutional Review Committee at Chung Shan Medical University, Taichung, Taiwan (approval number: CSMUH No. CS2-17018). RNA was extracted from these tissues then used for qRT-PCR analysis.

### 4.3. Quantitative Real-Time PCR (qRT-PCR)

Total RNA of each sample was extracted using a Trizol reagent (Invitrogen Life Technologies, Carlsbad, CA) and used to synthesize the first-strand cDNA using the SuperScript III first-strand synthesis kit system (Invitrogen Life Technologies, Carlsbad, CA, USA). All cDNA samples were diluted as a working template in qRT-PCR, which was carried out on an ABI StepOne TM Real-Time PCR System (Applied Biosystems, Foster City, CA, USA). The primer sequences used in this study were listed as follows: LINC00312: 5′-TGCCTTAAACCAGTTGTGCC-3′ and 5′-TTCATAGGCCCCTGTGCTAA-3′; and GAPDH: 5′-CTCATGACCACAGTCCATGC-3′ and 5′- TTCAGCTCTGGGATGACCTT-3′.

### 4.4. RNA Sequencing

To detect the differential expression of the transcriptome between normal and OSF tissues, we used a Trizol reagent to collect total RNA. A portion of the RNA isolate from each sample was submitted for quality assurance prior to RNA sequencing by the manufacturer of Genomics inc. Following mRNA enrichment, fragmentation, reverse transcription, library construction, and sequencing, the transcriptome was aligned according to the FPKM (fragments per kb of transcript per million mapped reads) values for detection of discrepancies in the transcript levels within cells.

### 4.5. Lentiviral-Mediated Silencing of LINC00312 and YBX1

The pLV-RNAi vector was obtained from Biosettia Inc. (Biosettia, San Diego, CA, USA). The shRNA sequence was designed and cloned using the single oligonucleotide RNAi technology following the manufacturer’s protocol. The target sequences for LINC00312 were listed as follows: Sh-LINC00312-1: 5′- AAAAGCTTGCTACATGAGACAATTTGGATCCAAATTGTCTCATGTAGCAAGC-3′; Sh- LINC00312-2: 5′- AAAAGCAGTAGTTCAAATCACAATTGGATCCAATTGTGATTTGAACTACTGC -3. Lentivirus production will be performed by cotransfection of a plasmid DNA mixture with a lentivector plus helper plasmids (VSVG and Gag-Pol) into HEK-293T cells (American Type Culture Collection, Manassas, VA, USA) using Lipofectamine 2000 (LF2000, Invitrogen, Carlsbad, CA, USA).

### 4.6. Collagen Gel Contraction Assay

Cells were suspended and mixed with a culture medium containing 2 mg/mL of collagen solution, Collagen (Cat. #C2124, Sigma-Aldrich, St. Louis, MO, USA). Next, the cell-gel mixture was incubated at 37 °C for 2 h in a 24-well-plate. After polymerization of the collagen gels, the gels were dislodged from the edge of the dish using a sterile spatula followed by further incubation for 48 h. The image of the gels was photographed and measured using the ImageJ software (NIH, Bethesda, MD, USA) to calculate their areas.

### 4.7. Western Blot Analysis

Cell lysates were subjected to SDS-PAGE and transferred to the PVDF membrane (Amersham, Arlington Heights, IL, USA) by a wet-transfer. Membranes were probed with primary antibodies overnight at 4 °C. The primary antibodies used included anti-α-SMA (Cat. #A5228), anti-COL1A1 (Cat. #C2456), anti-Smad2, and anti-phosphorylated Smad2 (Cat. #ZRB04953) (Santa Cruz Biotechnology, Inc., Santa Cruz, CA, USA). Following incubation of primary antibodies, the membranes were rinsed three times and incubated with corresponding secondary antibodies. The immunoreactive bands were developed using an ECL-plus chemiluminescence substrate (Perkin-Elmer, Waltham, MA, USA) and detected by the LAS-1000 plus Luminescent Image Analyzer (GE Healthcare, Piscataway, NJ, USA).

### 4.8. Transwell Migration and Invasion Assay

The cell migration capacity was measured using the transwell chambers (Corning, Acton, MA, USA). Cells were placed in the upper chamber with a 0.5% fetal bovine serum, and the lower chamber containing the higher serum as a chemoattractant. For the invasion assay, the membrane was coated with Matrigel (BD Pharmingen, NJ, USA). In the upper chamber, cells were seeded at a cell density of 1 × 10^5^ in a serum-free medium followed by 24 h of incubation. Cells were allowed to migrate to the lower surface of the membrane for 24 h at 37 °C and were stained with crystal violet. The number of cells in a total of five randomly selected fields were calculated.

### 4.9. Wound Healing Assay

Cells were seeded into a 6-well culture dish, and the confluent cells were mechanically wounded by passing a sterile 200 µL plastic pipette tip through the monolayer with one stroke. The rate of wound recovery (cell movement towards the center of the wound area) was monitored using an inverted phase-contrast microscope at 0 and 48 h.

### 4.10. Overexpression of LINC00312

LINC00312 cDNA was cloned into pLV-EF1a-MCS-IRES-Puro (Biosettia, Cat. No: cDNA-pLV01; San Diego, CA, USA). Lentivirus production was performed by cotransfection of a plasmid DNA mixture with a lentivector plus helper plasmids (VSVG and Gag-Pol) into HEK-293T cells (American Type Culture Collection). The pCDH1-MCS1-EF1-copGFP empty vector alone is utilized for the experimental control.

### 4.11. Statistical Analysis

SPSS Statistics version 13.0 was used for the statistical analysis. Student’s *t*-test or the ANOVA analysis with post-hoc Tukey HSD was used to determine the statistical significance of the differences among the experimental groups. *p* < 0.05 was considered statistically significant. Pearson’s and Spearman’s correlation were used to analyze the relationship between the expression of myofibroblast-related markers and LINC00312.

## 5. Conclusions

Our results demonstrated that the elevation of LINC00312 in OSF specimens contributed to the increased transdifferentiation of BMFs via YBX1. We observed a higher expression of LINC00312 and YBX1 in OSF tissues and there was a positive correlation between these two factors. YBX1 may exert its fibrosis function through the modulation of Smad2 and the LINC00312/YBX1 axis can function as a treatment target for OSF patients.

## Supplementary Materials

Supplementary materials can be found at https://www.mdpi.com/1422-0067/21/8/2979/s1.
