# Supplementary material for: LINC00312/YBX1 Axis Regulates Myofibroblast Activities in Oral Submucous Fibrosis
Source: Int J Mol Sci. 2020 Apr 23;21(8):2979. doi: 10.3390/ijms21082979 (PMC7215884; doi:10.3390/ijms21082979)
Supplement: Supplementary file 1 [file ijms-21-02979-s001.pdf]

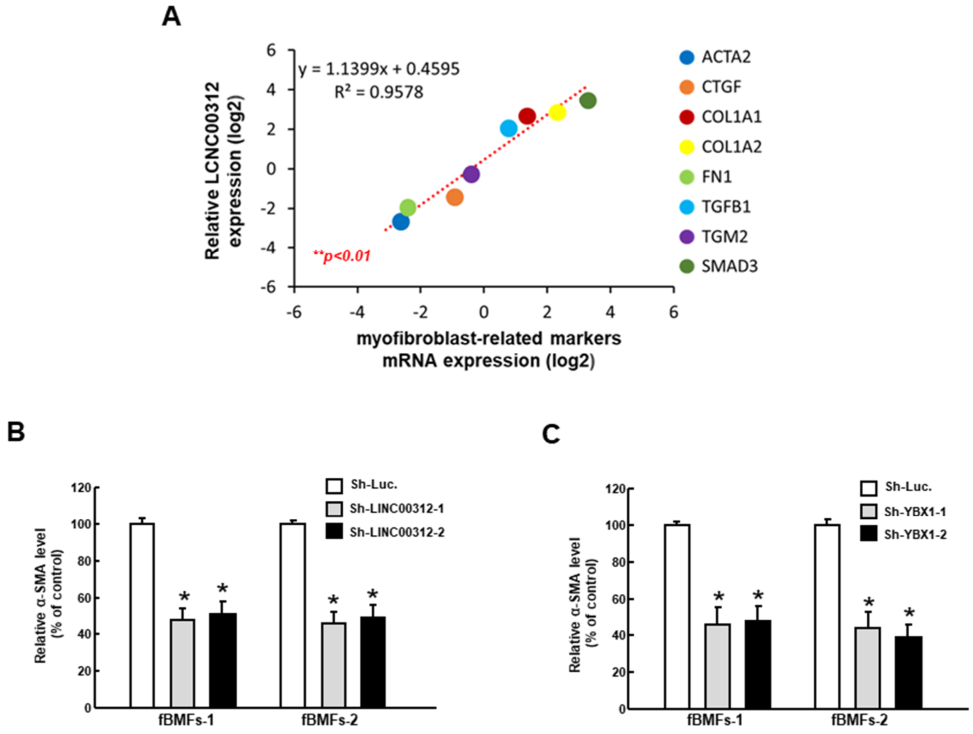

Supplementary Figure S1. (**A**) LINC00312 is positively correlated with myofibroblasts markers by Pearson correlation analysis. Levels of α -SMA expressions in fBMFs with LINC00312 (**B**) or YBX-1 (**C**) knockdown were examined by western blotting and measured by densitometer. The relative level of indicated protein expression was normalized against GAPDH and the control was set as 100%. Optical density values represent the mean ± SD. Data shown here are the mean±SD of three independent experiments. * $p < 0.05$ compared to Sh-Luc. group.
